# A Retrospective Study on the Effectiveness and Safety of Vancomycin versus Daptomycin in Hemodialysis Patients

**DOI:** 10.3390/antibiotics11060710

**Published:** 2022-05-25

**Authors:** Hideo Kato, Mao Hagihara, Mariko Kato, Yuka Yamagishi, Takumi Umemura, Nobuhiro Asai, Jun Hirai, Takuya Iwamoto, Hiroshige Mikamo

**Affiliations:** 1 Department of Clinical Infectious Diseases, Aichi Medical University, Nagakute 480-1195, Japan; katou.hideo.233@mail.aichi-med-u.ac.jp (H.K.); hagimao@aichi-med-u.ac.jp (M.H.); kato-mariko@med.mie-u.ac.jp (M.K.); y.yamagishi@mac.com (Y.Y.); umemuratakumi@gmail.com (T.U.); nobuhiro0204@gmail.com (N.A.); hiraichimed@gmail.com (J.H.); 2 Department of Pharmacy, Mie University Hospital, Tsu 514-8507, Japan; taku-iwa@med.mie-u.ac.jp; 3 Department of Clinical Pharmaceutics, Division of Clinical Medical Science, Mie University Graduate School of Medicine, Tsu 514-8507, Japan; 4 Department of Molecular Epidemiology and Biomedical Sciences, Aichi Medical University Hospital, Nagakute 480-1195, Japan

**Keywords:** vancomycin, daptomycin, hemodialysis patient, loading dose

## Abstract

Vancomycin or daptomycin is administered to hemodialysis patients infected with methicillin-resistant *Staphylococcus* and *Enterococcus* species. Although serious concerns regarding nephrotoxicity due to vancomycin have been raised, it might not be a critical issue in hemodialysis patients. Moreover, very few studies have investigated the effectiveness of vancomycin versus daptomycin in patients undergoing hemodialysis. Hence, we retrospectively evaluated the effectiveness and safety of vancomycin and daptomycin in patients undergoing hemodialysis. We investigated the following measures: mortality, clinical and microbiological effectiveness, and incidence of adverse events in hemodialysis patients who received vancomycin or daptomycin from 2014 to 2019. Moreover, we evaluated the covariates related to 30-day mortality. We found that 73 patients received vancomycin, while 34 received daptomycin for the treatment of infections due to methicillin-resistant *Staphylococcus aureus*, methicillin-resistant coagulase-negative *Staphylococci*, and *Enterococcus faecium*. Mortality after vancomycin treatment was significantly lower than daptomycin treatment (4.1% vs. 26.5%, *p* < 0.01). The clinical and microbiological effectiveness as well as the safety were not significantly different between the two treatments. Although daptomycin treatment with a loading dose was associated with lower mortality, the mortality of the treatment (8.3%) did not differ significantly compared to that of the vancomycin treatment (4.1%). Therefore, our findings suggest that vancomycin remains the first-line treatment for hemodialysis patients; however, a loading dose may be beneficial for patients receiving daptomycin.

## 1. Introduction

The absolute and relative risks of mortality from infections in hemodialysis patients have greatly increased due to their immunocompromised state, frequent exposure to the hospital environment, and tunneled catheters that allow the formation of biofilms [1,2]. Infection accounts for 12% to 36% of mortality in hemodialysis patients and is second only to cardiovascular disease as a cause of death [3,4]. The most common species detected in hemodialysis patients are *Staphylococcus*, including methicillin-resistant *Staphylococcus aureus* (MRSA) and methicillin-resistant coagulase-negative *Staphylococci* (MRCNS), which account for 69.1% of these microbes [5]. Moreover, it has been reported that infection mortality due to MRSA and MRCNS is 63.2% [5]. Therefore, appropriate antibiotic selection for infections due to MRSA and MRCNS in hemodialysis patients is an important factor for improving mortality.

Current guidelines recommend the use of vancomycin or daptomycin for the empirical treatment of MRSA infections in hemodialysis patients [6]. Although vancomycin is still considered the first-line treatment for MRSA infections owing to its bactericidal action, serious concerns regarding its safety profile, such as nephrotoxicity, have been raised [7]. However, this may not be a critical issue for hemodialysis patients. Moreover, very few studies have investigated the effectiveness of vancomycin versus daptomycin in patients undergoing hemodialysis. Hence, we retrospectively evaluated the effectiveness and safety of vancomycin and daptomycin in hemodialysis patients.

## 2. Results

### 2.1. Patients

**Figure 1:**
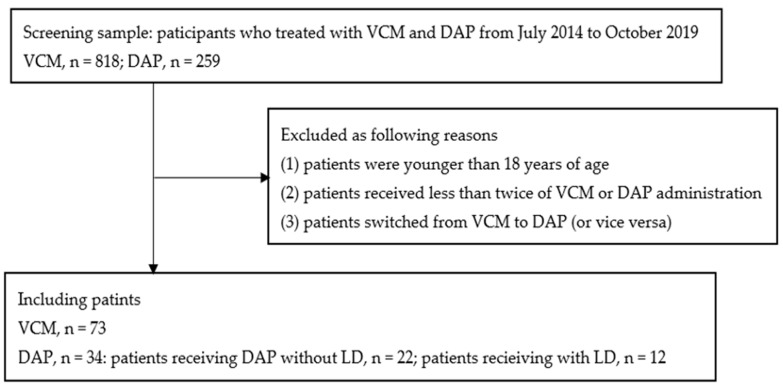
Flow diagram of study participants. DAP, daptomycin; LD, loading dose; VCM, vancomycin.

In total, 107 patients met the inclusion criteria during the study period, wherein 73 received vancomycin (vancomycin trough concentrations, 16.4 ± 4.4 mg/L) and 34 received daptomycin. Among the patients who received daptomycin, 12 received a loading dose (Figure 1).

The demographic and clinical characteristics stratified by therapeutic regimen are presented in Table 1. Comparing both treatments, the males and patients receiving a loading dose in the vancomycin group had a significantly higher rate than in the daptomycin group (vancomycin vs. daptomycin: males, 87.7% vs. 67.6%, *p* = 0.01; receiving a loading dose, 82.2% vs. 35.3%, *p* < 0.01). The others did not differ significantly between the two groups.

### 2.2. Microbiological Data

Vancomycin and daptomycin were administered to treat infections caused by MRSA, MRCNS, or *Enterococcus faecium* (Table 1). The most frequent isolates detected in this study were MRSA (vancomycin, 60.3%; daptomycin, 67.7%; *p* = 0.46), followed by MRCNS (31.5%; 29.4%; *p* = 0.83) and *E. faecium* (8.2%; 0%; *p* = 0.30). Regarding the minimum inhibitory concentration (MIC) distribution of these strains against vancomycin and daptomycin, the median MICs were 1.0 mg/L (range, 0.5–4.0 mg/L) and 1.0 mg/L (range, 0.25–2.0 mg/L), respectively. Moreover, one and two resistant isolates were detected in patients receiving vancomycin and daptomycin, respectively. However, vancomycin-resistant *Enterococcus* was not detected in the present study.

### 2.3. Clinical and Microbiological Effectiveness

The percentage of patients who reached a body temperature of <37.0 °C after the end of the treatment was 55.9% for vancomycin and 44.1% for daptomycin. The percentage of patients who reached CRP levels <60% of the baseline was 55.4% for vancomycin and 44.1% for daptomycin. There were no significant differences between vancomycin and daptomycin treatments (body temperature, *p* = 0.26; CRP level, *p* = 0.29; Table 2). Vancomycin showed lower mortality on days 14 (2.7% vs. 11.8%, *p* = 0.06) and 30 (4.1% vs. 26.5%, *p* < 0.01; Table 2) as compared to daptomycin. Especially, vancomycin in bacteremia and skin and soft tissue infections showed lower mortality on days 30 as compared to daptomycin (vancomycin vs. daptomycin: bacteremia, 6.7% vs. 27.8%, *p* = 0.04; skin and soft tissue infections, 3.0% vs. 25.0%, *p* = 0.02). In a subgroup of patients with bacteremia due to MRSA and *E. faecium*, there was no significant difference between the two groups (8.3% vs. 33.3%, *p* = 0.15). Microbiological cure rates showed no significant difference between both groups (78.6% vs. 77.7%, *p* = 0.94; Table 2).

### 2.4. Safety Evaluation

The safety data are listed in Table 3. According to the study criteria, elevated CK and eosinophil counts were observed in two and seven patients, respectively. No patient was diagnosed with eosinophilic pneumonia in this population, whereas four were diagnosed with allergies. Additionally, the total number of patients who were admitted with abnormalities in AST and ALT levels was ten and nine, respectively. However, the incidence of adverse events was not significantly different between the two treatments.

### 2.5. Comparison between the Survival and Non-Survival Groups

The mortality in our population was 11.2% (survival group, n = 95; non-survival group, n = 12). Table 4 shows the differences in the covariates related to 30-day mortality between the survival and non-survival groups. Receiving a loading dose of vancomycin or daptomycin was associated with a significant reduction in mortality (survival group vs. non-survival group, 72.6% vs. 25.0%, *p* < 0.01). Receiving the loading dose of vancomycin did not improve mortality (82.9% vs. 66.7%, *p* = 0.472), whereas that of daptomycin showed a tendency to reduce mortality (44.0% vs. 11.1%, *p* = 0.08). Moreover, microbiological effect was higher in the survival group than in the non-survival group (81.1% vs. 55.6%, *p* = 0.08). The other measures did not significantly differ between the two groups. In the logistic analysis (Table 4), covariates significantly related to the mortality were identified: receiving a loading dose (*p* < 0.01, OR 7.96, 95% CI 2.00–31.72), receiving a loading dose of daptomycin (*p* = 0.10, OR 6.29, 95% CI 0.68–58.11), and microbiological effectiveness (*p* = 0.09, OR 3.43, 95% CI 0.81–14.44).

### 2.6. Clinical Effectiveness and Safety in Hemodialysis Patients Receiving Vancomycin and Daptomycin Treatments with or without a Loading Dose

Patients receiving a daptomycin treatment without a loading dose were 22, while those with a loading dose were 12. Regarding clinical effectiveness, the 30-day mortality was significantly different among the three groups; the daptomycin treatment with a loading dose showed a lower mortality than that without (vancomycin treatment vs. daptomycin treatment without the loading dose vs. daptomycin treatment with the loading dose, 4.1% vs. 63.4% vs. 8.3%, *p* < 0.01; Table 5). The other groups did not differ significantly from each other. Regarding safety, there were no significant differences among the three groups (Table 6).

## 3. Discussion

To the best of our knowledge, this is the first study to compare the effectiveness and safety of vancomycin and daptomycin in patients undergoing hemodialysis. In our study, the vancomycin treatment improved mortality without significant adverse events compared with the daptomycin treatment. Moreover, the loading dose, especially that of daptomycin, was one of the covariates related to mortality, which was reduced to 8.3%. Therefore, vancomycin remains as the first-line treatment, while a loading dose of daptomycin may be beneficial in hemodialysis patients.

To date, studies in various populations have investigated and compared the effectiveness and safety of vancomycin and daptomycin [8,9]. A recent meta-analysis indicated that daptomycin was better tolerated than vancomycin in the treatment of MRSA infections without lung involvement [10]. On the other hand, a systematic review of hemodialysis patients reported that there were no studies evaluating the incidence of adverse events attributable to vancomycin [11]. Japanese studies, including the present one, have shown that hemodialysis patients do not frequently develop adverse events caused by vancomycin [12]. These observations highlight the higher tolerability of vancomycin in hemodialysis patients than in those with residual renal function.

In a meta-analysis, the risk of mortality failed to show a statistically significant difference between vancomycin and daptomycin for the treatment of MRSA infections in all patients [10]. However, it has been reported that the clinical failure rate of patients with impaired renal function is lower with vancomycin than with daptomycin [13,14]. Moreover, the present study of hemodialysis patients indicated that vancomycin significantly reduced mortality as compared to daptomycin. Therefore, vancomycin may be more effective than daptomycin in patients with impaired renal function, especially hemodialysis patients. In contrast, there were no significant differences in the clinical and microbiological effectiveness between vancomycin and daptomycin treatments. Moreover, the levels of inflammatory markers before treatment were not significantly different between the two groups. Therefore, the reduction in mortality may be associated with an improvement in the early clinical response. However, the present study did not design to measure the blood concentration of daptomycin even though daptomycin requires a considerable time to achieve steady-state concentrations because of half-life in hemodialysis patients. Further well-designed studies are needed to validate the effectiveness and safety of vancomycin versus daptomycin. Moreover, although caution is advised since the benefits of vancomycin for hemodialysis patients may depend on the MIC, the present as well as other studies [15,16] have found no association between antibiotic treatment and mortality.

A loading dose regimen has been recommended to rapidly reach an effective therapeutic concentration as well as to optimize pharmacokinetic and pharmacodynamic indicators [17,18,19,20]. However, a recent meta-analysis reported that a loading dose of vancomycin did not reduce mortality or improve microbiological effectiveness [21]. In contrast, we previously found in an in vivo study that a loading dose of daptomycin exhibited a higher microbiological activity [22]. Retrospective studies showed that similar treatments also improved clinical symptoms at an early stage [17,23]. In the present study, daptomycin treatment without the loading dose showed a significantly higher mortality than the vancomycin treatment (*p* < 0.01), while the mortality in the patients receiving daptomycin treatment with the loading dose was equivalent with those of the vancomycin treatment (*p* = 0.52). Therefore, daptomycin treatment with the loading dose appears to be effective for hemodialysis patients. However, further large-scale studies are needed since the sample size is limited.

Recent practical guideline has recommended a high dose of daptomycin [6]. However, the present retrospective study did not include patients receiving a high dose of daptomycin since previous studies of patients with renal impairment reported that the high dose was associated with a high incidence of creatinine kinase elevation [24]. Therefore, further studies to compare a normal dose with a high dose of daptomycin are needed to validate the effectiveness of a high dose of daptomycin.

Several considerations should be made when interpreting our results. First, this was a non-randomized, single-center, retrospective study. We were unable to investigate the degree of disease severity owing to a lack of data. Selection bias could have been present, and missing data may have influenced the results. Thirty-four patients were treated with daptomycin throughout the study period, and patients receiving a loading dose of daptomycin were 12. The quality of the study is limited by its small sample size. It should be noted that patients, in whom MRSA, MRCNS, or *E. faecium* were detected, were included in our study. Although we were not able to evaluate the microbiological effectiveness in 22.4% of the patients, more detailed microbiological data regarding the MIC values were reported.

## 4. Patients and Methods

### 4.1. Patient Population

We retrospectively reviewed the medical records of hemodialysis patients treated with vancomycin or daptomycin at the Aichi Medical University Hospital from July 2014 to October 2019. Patients were excluded from the study for the following reasons: (1) patients were younger than 18 years of age, (2) patients received less than twice of vancomycin or daptomycin administration, and (3) patients switched from vancomycin to daptomycin (or vice versa). The study was reviewed and approved by the ethics committee of the Aichi Medical University (No. 2020-058).

### 4.2. Treatment Regimen

Vancomycin was administered to achieve a target trough of 10–20 mg/L on the day of the second hemodialysis according to Japanese therapeutic drug monitoring guidelines [25]. Daptomycin was administered thrice per week according to daptomycin prescription information (4–6 mg/kg/day). Some of the patients received a dose of 4–6 mg/kg daptomycin thrice per week with a loading dose (>8 mg/kg) on day 1 based on our previous study results [17,23].

### 4.3. Data Collection

At least 3 days before the start of treatment, treatment data, including patient demographics, hospitalization history, source of infection, and laboratory data, were retrospectively collected through chart review. The clinical outcomes and antibiotic-related adverse reactions were recorded for each patient.4.4. Microbiological Data

Identification and susceptibility tests for the isolated organisms were conducted at the Department of Laboratory at Aichi Medical University Hospital. All isolates were susceptibility-tested using the broth microdilution method as described by the Clinical and Laboratory Standards Institute (CLSI) [26]. Vancomycin-resistant pathogens were defined as isolates with an MIC of >4 mg/L for *Staphylococcus* and >8 mg/L for *Enterococcus*. Daptomycin-resistant pathogens were defined as isolates with an MIC > 2 mg/L for *Staphylococcus* and >4 mg/L for *Enterococcus*. For the purpose of this study, all specimens (blood, wound, abscess, pus, or tissue) cultured at the microbiology laboratory during the study periods were included.

### 4.4. Clinical and Microbiological Effectiveness

The evaluation was performed in accordance with previous studies [17,22]. Data regarding inflammatory markers of body temperature and C-reactive protein (CRP) level at least 3 days before the start (baseline level) and after the end of antibiotic treatment were collected. For clinical effectiveness, we evaluated the percentages of patients who reached a body temperature <37.0 °C and CRP <60% of the baseline level. Survival was defined as survival at 14 and 30 days after antibiotic treatment. A microbiological cure was defined as effective when bacteria disappeared during and after antibiotic treatment.

### 4.5. Safety Evaluation

We evaluated abnormality with CTCAE version 4.03 the Common Terminology Criteria for Adverse Events (CTCAE). The abnormality was defined as follows: aspartate aminotransferase (AST), >2 × upper baseline (35 U/L); alanine aminotransferase (ALT), >2 × upper baseline (30 U/L); creatinine kinase (CK), >1.3 × upper baseline (200 U/L); and eosinophil granulocyte count, >500/μL. In addition, we assessed the onset of eosinophilic pneumonia and rash (allergies).

### 4.6. Evaluation of Covariate Related to 30-Day Mortality

The following factors were compared between survival and non-survival groups: loading dose, initial vancomycin trough concentrations, microbiological effectiveness, bacteremia, and detection of vancomycin- or daptomycin-resistant pathogens, MRSA, MRCNS, and *E. faecium*. Moreover, logistic regression analysis was performed to determine the odds ratio (OR) for 30-day mortality. Factors showing a *p*-value of 0.1 were considered candidate predictors significantly related to mortality.

### 4.7. Evaluation of a Loading Dose of Daptomycin for Clinical Effectiveness and Safety

Previously, we revealed that receiving a loading dose of daptomycin was a significant covariate for mortality in hemodialysis patients [23]. However, a logistic regression analysis showed that receiving a loading dose of daptomycin was identified as a positive covariate related to mortality. Thus, we compared the influence of daptomycin treatment with a loading dose of vancomycin and daptomycin treatment without a loading dose on clinical and microbiological outcomes in hemodialysis patients.

### 4.8. Statistical Analysis

The data regarding the clinical characteristics of the patients were expressed as median values (minimum–maximum). Statistical significance was evaluated using the chi-square test for categorical data and the unpaired t-test for continuous data. Statistical analysis was performed using JMP, version 10.0 (SAS, Tokyo, Japan). A *p*-value of <0.05 was required to achieve statistical significance.

## 5. Conclusions

In conclusion, our study indicated that vancomycin significantly reduced mortality in hemodialysis patients as compared to daptomycin. Although daptomycin treatment with a loading dose was associated with lower mortality, the mortality of the treatment did not differ significantly compared with that of vancomycin. Therefore, our findings suggest that vancomycin remains as the first-line treatment for hemodialysis patients, but a loading dose may be beneficial for patients receiving daptomycin.

## Figures and Tables

**Table 1:** Clinical characteristics of the registered patients.

	VCM	DAP	*p* Value
Gender (male/female)	64/9	23/11	0.01
Age (years)	71 (46–85)	73 (46–91)	0.23
Body weight (kg)	56.7 (32.4–115.5)	50.7 (35.0–100.0)	0.29
Duration of therapy (days)	13 (3–74)	13 (5–52)	0.09
Dosage on day 1 (mg/kg)	18.9 (5.4–30.9)	6.4 (3.7–14.0)	-
Receiving a loading dose (%)	82.2 (60/72)	35.3 (12/34)	<0.01
WBC (×10^3^/μL)	8.2 (2.1–26.6)	8.2 (1.0–27.8)	0.44
Eosinophil granulocytes (%)	2.0 (0.2–15.0)	2.0 (0.1–21.2)	0.49
Serum creatinine (mg/dL)	6.15 (1.70–13.45)	5.08 (1.34–12.29)	0.08
eGFR (mL/min/1.73 m^2^)	8 (3–30)	9 (3–37)	0.06
AST (U/L)	18 (8–5537)	21 (7–85)	0.51
ALT (U/L)	9 (1–2905)	10 (3–40)	0.52
CK (U/L)	40 (2–2794)	52 (3–591)	0.45
CRP (mg/dL)	7.38 (0.03–33.13)	7.56 (0.52–34.94)	0.43
Body temperature (°C)	37.1 (36.3–40.3)	37.3 (36.5–40.2)	0.27
Detected isolates			
MRSA (%)	60.3 (44/73)	67.7 (23/34)	0.46
MRCNS (%)	31.5 (23/73)	29.4 (10/34)	0.83
*E. faecium* (%)	8.2 (6/73)	2.9 (1/34)	0.30
Resistant pathogen (%)	1.4 (1/73)	5.9 (2/34)	0.19
Comrmodity			
Cancer	11.0 (8/73)	14.7 (5/34)	0.58
Diabates	31.5 (23/73)	32.4 (11/34)	0.85
Types of infection			
Bacteremia (%)	41.1 (30/73)	52.9 (18/34)	0.25
SSTIs (%)	45.2 (33/73)	47.1 (16/34)	0.86
Pneumonia (%)	9.6 (7/73)	0 (0/34)	0.07
UTI (%)	4.1 (3/73)	0 (0/34)	0.23

The chi-square and unpaired t-tests were used for analyzing categorical and continuous data, respectively. All data, except gender are shown as the median (minimum–maximum). VCM, vancomycin (n = 73); DAP, daptomycin (n = 34). ALT, alanine aminotransferase; AST, alanine aminotransferase; CK, creatinine kinase; CRP, C-reactive protein; eGFR, estimated glomerular filtration rate; MRCNS, methicillin-resistant coagulase-negative staphylococci; MRSA, methicillin-resistant Staphylococcus aureus; SSTIs, skin and soft tissue infections; UTI, urinary tract infection; WBC, white blood cell.

**Table 2:** Clinical and microbiological effectiveness of the registered patients.

	VCM	DAP	*p*-Value
BT of <37.0 °C (%)	55.9 (38/68)	44.1 (15/34)	0.26
CRP of <60% (%)	55.4 (36/65)	44.1 (15/34)	0.29
14-day mortality (%)	2.7 (2/73)	11.8 (4/34)	0.06
30-day mortality (%)	4.1 (3/73)	26.5 (9/34)	<0.01
Microbiological effectiveness (%)	78.6 (44/56)	77.7 (21/37)	0.94

Chi-square test for categorical data. VCM, vancomycin; DAP, daptomycin. BT, body temperature; CRP, C-reactive protein.

**Table 3:** Safety data of the registered patients.

	VCM	DAP	*p*-Value
Increased AST level (%)	9.1 (6/66)	11.8 (4/34)	0.67
Increased ALT levels (%)	9.1 (6/66)	8.8 (3/34)	0.97
Increased blood CK level (%)	2.3 (1/43)	3.3 (1/30)	0.80
Increased eosinophil count (%)	11.4 (4/35)	11.5 (3/26)	0.99
Onset of eosinophilic pneumonia (%)	0 (0/73)	0 (0/34)	-
Rash (%)	4.1 (3/73)	2.9 (1/34)	0.77

Chi-square test for categorical data. VCM, vancomycin; DAP, daptomycin. AST, aspartate aminotransferase; ALT, alanine aminotransferase; CK, creatinine kinase.

**Table 4:** Comparison of survival and non-survival groups.

	Survival	Non-Survival	*p*-Value ^a^	*p*-Value ^b^, OR, 95%CI
Receiving a loading dose (%)	72.6 (69/95)	25.0 (3/12)	<0.01	<0.01, 7.96, 2.00–31.72
Receiving a loading dose of daptomycin (%)	44.0 (11/25)	11.1 (1/9)	0.08	0.10, 6.29, 0.68–58.11
Receiving a loading dose of vancomycin (%)	82.9 (58/70)	66.7 (2/3)	0.47	0.49
Initial VCM trough concentrations (mg/L)	16.3 ± 4.4	15.3 ± 6.6	0.69	0.69
Microbiological effectiveness (%)	81.1 (60/74)	55.6 (5/9)	0.08	0.09, 3.43, 0.81–14.44
Bacteremia (%)	43.2 (41/95)	58.3 (7/12)	0.32	0.32
Detected isolates				
Resistant pathogen (%)	2.1 (2/95)	8.3 (1/12)	0.22	0.25
MRSA (%)	62.1 (59/95)	66.7 (8/12)	0.76	0.76
MRCNS (%)	30.5 (29/95)	33.3 (4/12)	0.84	0.84
*E. faecium* (%)	7.4 (7/95)	0 (0/12)	0.33	0.97

^a^ Chi-square test for categorical data and unpaired t-test for continuous data. ^b^ Logistic regression analysis. Data on initial vancomycin trough concentrations are presented as mean ± standard deviation). Survival, patients who were alive on day 30 (n = 95); non-survival, patients who died by day 30 (n = 13). MRCNS, methicillin-resistant coagulase-negative *Staphylococci*; MRSA, methicillin-resistant *Staphylococcus aureus*; VCM, vancomycin.

**Table 5:** Clinical and microbiological effectiveness of patients receiving vancomycin and daptomycin treatments with or without a loading dose.

	VCM	DAP without LD	DAP with LD	*p*-Value
BT of <37.0 °C (%)	55.9 (38/68)	40.9 (9/22)	50.0 (6/12)	0.47
CRP of <60% (%)	55.4 (36/65)	40.9 (9/22)	50.0 (6/12)	0.50
14-day mortality (%)	2.7 (2/73)	13.6 (3/22)	8.3 (1/12)	0.14
30-day mortality (%)	4.1 (3/73)	36.4 (8/22)	8.3 (1/12)	<0.01
Microbiological effectiveness (%)	78.6 (44/56)	76.5 (13/17)	80.0 (8/10)	0.97

Chi-square test for categorical data. VCM, patients receiving vancomycin; DAP without LD, patients receiving a thrice-per-week dose according to daptomycin prescription information (4–6 mg/kg/day); DAP with LD, patients receiving a dose of 4–6 mg/kg daptomycin thrice per week with a loading dose (>8 mg/kg) on day 1. BT, body temperature; CRP, C-reactive protein; DAP, daptomycin; LD, loading dose; VCM, vancomycin.

**Table 6:** Safety data of patients receiving vancomycin and daptomycin treatments with or without a loading dose.

	VCM	DAP without LD	DAP with LD	*p*-Value
Increased AST level (%)	9.1 (6/66)	4.5 (1/22)	25.0 (3/12)	0.15
Increased ALT level (%)	9.1 (6/66)	4.5 (1/22)	16.7 (2/12)	0.50
Increased blood CK levels (%)	2.3 (1/43)	0 (0/19)	9.1 (1/11)	0.33
Increased eosinophil granulocyte count (%)	11.4 (4/35)	12.5 (2/16)	10.0 (1/10)	0.98
Onset of eosinophilic pneumonia (%)	0 (0/73)	0 (0/22)	0 (0/12)	-
Rash (%)	4.1 (3/73)	5.6 (1/22)	0 (0/12)	0.77

Chi-square test for categorical data. VCM, patients receiving vancomycin; DAP without LD, patients receiving a thrice-per-week dose according to daptomycin prescription information (4–6 mg/kg/day); DAP with LD, patients receiving a dose of 4–6 mg/kg daptomycin thrice per week with a loading dose (>8 mg/kg) on day 1. AST, aspartate aminotransferase; ALT, alanine aminotransferase; CK, creatinine kinase; DAP, daptomycin; LD, loading dose; VCM, vancomycin.

## Data Availability

All data are available in the paper.
